# Linked patterns of symptoms and cognition with brain controllability in major depressive disorder

**DOI:** 10.1192/j.eurpsy.2023.904

**Published:** 2023-07-19

**Authors:** Q. Li, Y. Zhao, Y. Wang, F. Long, Q. Gong, F. Li

**Affiliations:** Huaxi MR Research Center (HMRRC), Radiology, West China Hospital of Sichuan University, Chengdu, China

## Abstract

**Introduction:**

Major depressive disorder (MDD) is characterized by both clinical symptoms and cognitive deficits. Prior studies have typically examined either symptoms or cognition correlated with brain measures, thus causing a notable paucity of stable brain markers that capture the full characteristics of MDD. Brain controllability derived from newly proposed brain model integrating both metabolism (energy cost) and dynamics from a control perspective has been considered as a sensitive biomarker for characterizing brain function. Thus, identifying such a biomarker of controllability related to both symptoms and cognition may provide a promising state monitor of MDD.

**Objectives:**

To assess the associations between two multi-dimensional clinical (symptoms and cognition) and brain controllability data of MDD in an integrative model.

**Methods:**

Sparse canonical correlation analysis (sCCA) was used to investigate the association between brain controllability at a network level and both clinical symptoms and cognition in 99 first-episode medication-naïve patients with MDD. The potential mediation effect of cognition on relationship between controllability and symptoms was also tested.

**Results:**

Average controllability was significantly correlated with both symptoms and cognition (*r*_mean_=0.54, *P*_Bonferroni_=0.03). Average controllability of dorsal attention network (DAN) (*r*=0.46) and visual network (*r*=0.29) had the highest correlation with both symptoms and cognition. Among clinical variables, depressed mood (*r* =-0.23) , suicide(*r* =-0.25), work and activities(*r* =-0.27), gastrointestinal symptoms (*r* =-0.25) were significantly negatively associated with average controllability, while cognitive flexibility (*r*=0.29) was most strongly positively correlated with average controllability. Additionally, cognitive flexibility fully mediated the association between average controllability of DAN and depressed mood (indirect effect=-0.11, 95% CI [-0.18, -0.04], *P*=0.001) in MDD.

**Conclusions:**

Brain average controllability was correlated with both clinical symptoms and cognition in first-episode medication-naïve patients with MDD. The results suggest that average controllability of DAN and visual network reached high associations with clinical variates in MDD, thus these brain features may serve as stable biomarkers to control the brain functional states transitions to be relevant to cognitions deficits and clinical symptoms of MDD. Additionally, altered average controllability of DAN in patients could induce impairment of cognitive flexibility, and thus cause severe depressed mood, indicating that controllability of DAN may be a potential intervention target for alleviating depressed mood through improving cognitive flexibility in MDD.

**Disclosure of Interest:**

None Declared

